# Quantitative evaluation of dosimetric uncertainties in electron therapy by measurement and calculation using the electron Monte Carlo dose algorithm in the Eclipse treatment planning system

**DOI:** 10.1002/acm2.13478

**Published:** 2021-11-25

**Authors:** Imad Ali, Nesreen Alsbou, Salahuddin Ahmad

**Affiliations:** ^1^ Department of Radiation Oncology University of Oklahoma Health Sciences Center Oklahoma City Oklahoma USA; ^2^ Department of Engineering and Physics University of Central Oklahoma Edmond Oklahoma USA

**Keywords:** bremsstrahlung, commissioning data, dose calculation algorithm, dosimetric uncertainties, heterogeneity correction, image artifacts

## Abstract

In the electron beam radiation therapy, customized blocks are mostly used to shape treatment fields to generate conformal doses. The goal of this study is to investigate quantitatively dosimetric uncertainties associated with heterogeneities, detectors used in the measurement of the beam data commissioning, and modeling of the interactions of high energy electrons with tissue. These uncertainties were investigated both by measurements with different detectors and calculations using electron Monte Carlo algorithm (eMC) in the Eclipse treatment planning system. Dose distributions for different field sizes were calculated using eMC and measured with a multiple‐diode‐array detector (MapCheck2) for cone sizes ranging from 6 to 25 cm. The dose distributions were calculated using the CT images of the MapCheck2 and water‐equivalent phantoms. In the umbra region (<20% isodose line), the eMC underestimated dose by a factor of 3 for high energy electron beams due to lack of consideration of bremsstrahlung emitted laterally that was not accounted by eMC in the low dose region outside the field. In the penumbra (20%–80% isodose line), the eMC overestimated dose (40%) for high energy 20 MeV electrons compared to the measured dose with small diodes in the high gradient dose region. This was mainly due to lack of consideration of volume averaging of the ion chamber used in beam data commissioning which was input to the eMC dose calculation algorithm. Large uncertainties in the CT numbers (25%) resulted from the image artifacts in the CT images of the MapCheck2 phantom due to metal artifacts. The eMC algorithm used the electron and material densities extracted from the CT numbers which resulted large dosimetric uncertainties (10%) in the material densities and corresponding stopping power ratios. The dose calculations with eMC are associated with large uncertainties particularly in penumbra and umbra regions and around heterogeneities which affect the low dose level that cover nearby normal tissue or critical structures.

## 1 INTRODUCTION

Electron radiation therapy is the most common technique in the treatment of skin cancer and superficial lesions.^1^, ^2^ It is often combined with photon therapy to provide boost doses for head and neck and breast tumors.^3^, ^4^, ^5^ Usually a customized single electron field with a cutout for skin cancer is used to produce conformal dose superficially without excessive doses to normal tissue or critical structures beneath the tumor. The dose calculation for electrons is usually performed using simple methods based on empirical modeling with lookup tables for scatter factors and depth dose curves that are dependent on field sizes and beam energies.^6^, ^7^ Sophisticated dose calculation algorithms based on modeling of the electron interactions with tissue developed by different vendors are also available.^8^, ^9^, ^10^ These algorithms include pencil beam convolution^11^, ^12^, ^13^ and Monte Carlo simulation techniques^14^, ^15^, ^16^ which calculate dose distributions in three dimensions using CT images obtained during patient simulations. The electron dose distributions are used for more accurate plan evaluation. They are often combined with the photon dose distributions to produce sum doses which are used to evaluate the tumor dose coverage and sparing of nearby normal tissues.

Most of the commercially available electron dose calculation algorithms use the CT number values from the patient CT images to extract the mass stopping power ratios and correct the heterogeneity of the different tissues.^9^, ^17^, ^18^, ^19^, ^20^ However, heterogeneity correction in electron dosimetry is complicated by finding accurate stopping power ratios.^21^, ^22^ The CT number values represent more electron density of the material, they do not provide accurate values for the Z‐composition and the material density. The lack of appropriate experimental benchmarks for testing and dose verification of electron Monte Carlo (eMC) dose calculation algorithms is associated with large uncertainties in calculating absolute doses.^23^, ^24^ Another issue in electron dosimetry is associated with accurate modeling of the bremsstrahlung resulting from the interaction of high energy electron beams with the medium.^25^, ^26^, ^27^, ^28^ Furthermore, there are few studies that reported about well‐established approaches to verify the commissioning data and process of electron dose calculation algorithms in comparison with photon dosimetry. The electron commissioning data are usually specified and measured using vendor recommendation with commercially available ion chambers and data acquisition systems. Uncertainties are often associated with the use of appropriate setup and detectors used in collecting the commissioning data and process of electron dose calculation algorithms.^9^, ^29^, ^30^ Furthermore, few studies have investigated in a systematic approach the issues in small electron fields compared to small field photon dosimetry.^29^, ^30^, ^31^, ^32^ The small field electron dosimetry is associated with issues such as lack of charge particle equilibrium, source occlusion, and volume averaging of the dose measurement by large detectors similar to small field photon dosimetry.^33^, ^34^

In this work, the MapCheck2 was used to verify the dose distributions calculated by the eMC algorithm after the commissioning process of the Eclipse treatment planning system. Two‐dimensional electron dose distributions were measured with the MapCheck2 which is a multiple‐diode‐array detector used often in photon dosimetry.^35^ The following dosimetric uncertainties were identified and quantified in this study that were not addressed thoroughly in previous works: (a) modeling of bremsstrahlung dose in the umbra region by the eMC dose calculation algorithm, (b) measurement of commissioning data with large detectors such as ion chambers in the penumbra region, and (c) heterogeneity correction in the eMC algorithm.

## 2 MATERIALS AND METHODS

### 2.1 Electron Monte Carlo dose calculation in eclipse

The commissioning beam data were measured with the CC13 ion chamber (IBA Dosimetry, 315 Stage Post Dr. # 110, Memphis, TN, USA) and were input in the Eclipse treatment planning system (Varian Medical Systems, Inc., Palo Alto, CA, USA) to model the eMC algorithm.^16^, ^20^ The commissioning data included the following: (a) measurement of the depth dose curves for the cones with sizes ranging from 6 × 6 to 25 × 25 cm^2^ in water, (b) the scatter factors at depths of maximum dose for each energy for the different cones relative to the reference cone of 10 × 10 cm^2^, and (c) measurement of the fluence profiles in air for a large field of 40 × 40 cm^2^ for each electron energy. The eMC was then used for dose calculation with electron beams (6, 9, 12, 16, and 20 MeV) using different cones (6 × 6, 10 × 10, 15 × 15, 20 × 20, and 25 × 25 cm^2^) and source‐to‐surface distances (SSD) (104–110 cm) covering a wide clinical range. The dose distributions for the previous fields were calculated on a 40 × 40 × 40 cm^3^ water phantom that was generated in the Eclipse treatment planning system. Furthermore, the dose distributions were calculated on the CT images of the MapCheck2 phantom in order to investigate heterogeneity effects from high‐Z materials that make up the MapCheck2 phantom (Sun Nuclear, Melbourne, FL, USA).^35^, ^36^ Helical CT images were acquired for the MapCheck2 phantom using a thorax imaging technique with a CT simulator (GE Discovery‐CT‐590RT, General Electric Healthcare, Milwaukee, WI, USA) using a pitch of 1.375, 2.5 mm slice thickness, 120 kVp, and 440 mA. The CT images provided the CT number values for eMC that were used to consider heterogeneity of the MapCheck2 phantom in the calculated dose distributions. The eMC algorithm uses two models: (a) an initial phase space model and (b) a transport model. The phase space model represents the electron interactions with scattering foils, ion chambers, primary and secondary collimators through the treatment head of the linear accelerator. The transport model implements a macro‐Monte Carlo approach using a local‐to‐global dose method which incorporates conventional Monte Carlo simulation of electron transport in local geometry for tissue‐equivalent materials of relevant energies. The transport calculation where the electrons are transported through the medium in macroscopic steps is performed and saved in probability distribution functions (PDFs) using EGSnrc code^30^, ^37^ for dose calculation in a global geometry. The highest energy electron is considered the primary particle while other energy electrons and bremsstrahlung photons are considered as secondary particles. The PDFs contain position, direction, and energy information of the emerging particle. They are obtained for physical parameters in combination with five different materials (air, lung phantom, water, Lucite, and solid bone phantom), 30 incident energy values ranging from 0.2 to 25 MeV, and five spheres with radii ranging from 0.5 to 3 mm. In this study, 2.5 mm grid size was used for dose calculation, nearly 40 million primary particles were simulated, the relative uncertainty of the mean dose was 4.89%, and a 3D Gaussian smoothing technique was used with medium smoothing level of 2% accuracy as listed in Table 1.

**TABLE 1:** Electron dose calculation parameters using the electron Monte Carlo (eMC) algorithm in the Eclipse treatment planning system

Grid size	2.5 mm
Mean dose accuracy	4.89%
Random generator seed number	40 million particles
Smoothing method	3D Gaussian smoothing
Smoothing level	2% accuracy
Energy levels	30 incident energy levels (0.2–2.5 MeV)
Heterogeneity correction	5 materials (air, lung phantom, water, Lucite, and solid bone phantom)
Scoring method	5 spheres with radii ranging from 0.5 to 3 mm

### 2.2 Experimental setup and dose measurement

**FIGURE 1:**
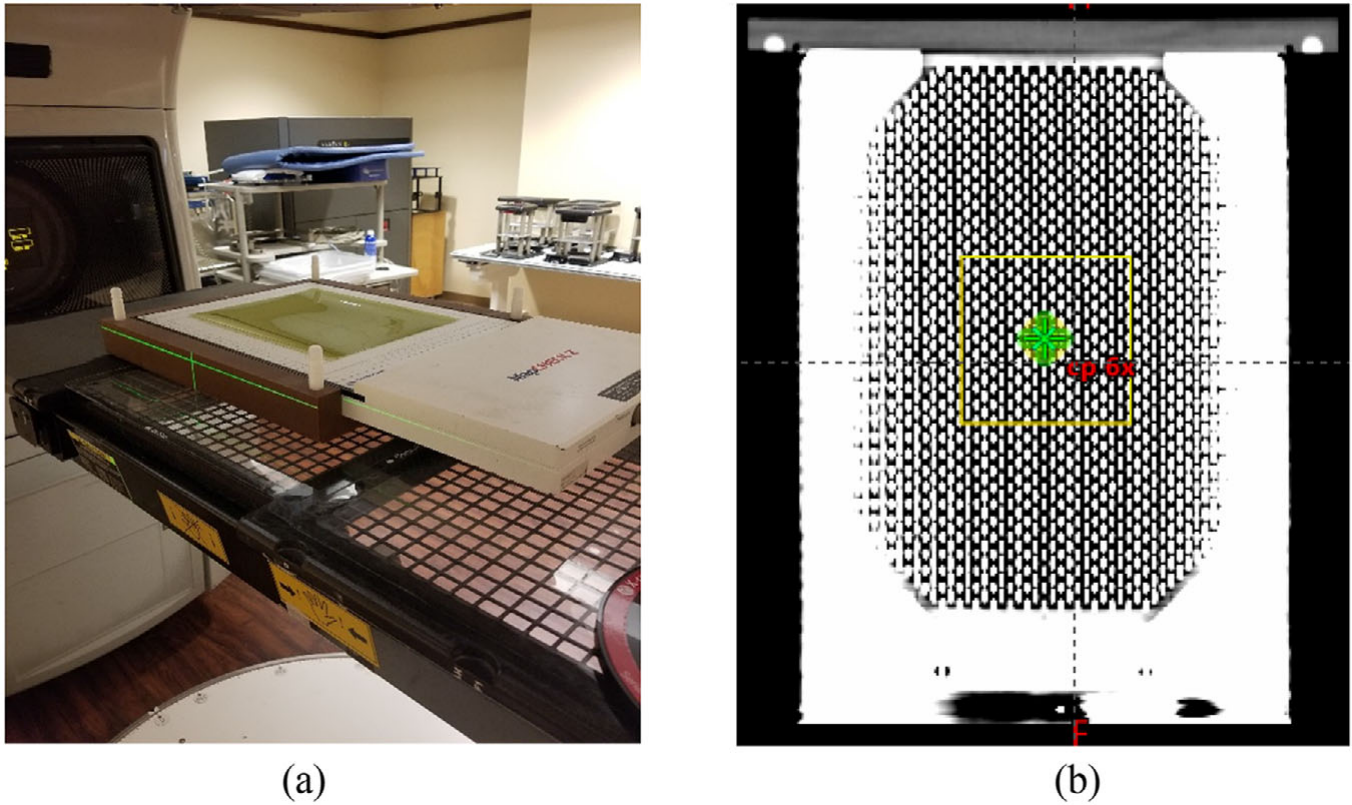
(a) MapCheck2 phantom setup without upper phantom encapsulation used to measure the 2D dose distributions. (b) Coronal view of the CT images of the MapCheck2 phantom

The treatment plans that were generated with eMC algorithm for different cones and energies were delivered using a Varian Trilogy machine (Varian Medical Systems, Inc.). The 2D dose distributions were measured using MapCheck2 where the upper phantom encapsulation was removed to measure the shallowest dose at a depth of 2.0 cm with phantom casing surrounding the diode‐array as shown in Figure 1a. The MapCheck2 phantom is made from solid water phantom, the effective layer is made from diodes that are made from silicon and solid‐state materials, and the encapsulation is made from a copper alloy surrounded by air cavities. It is made from 1527 diode detectors arranged in a 2D array with an effective area of 26 × 32 cm^2^ with an active detection area of 0.48 × 0.48 mm^2^ for each individual diode. The distance between the diodes is 10 mm in the rows and columns of the array detector. However, the position of the diodes in the different rows is offset from one another where the minimal diagonal distance between two nearby detectors is 7 mm. Although the spatial resolution of the MapCheck2 of the measured dose distributions is limited by the spacing between the diodes, the effective radiation detection resolution of each diode is about 0.48 mm which provides high position resolution dose gradient measurements in the penumbra locally at the site of the individual detectors. The diodes were located physically at 12 mm depth encapsulated with high‐Z materials that corresponded to water‐equivalent thickness of 20.0 mm for high energy photon and electron beams.

The MapCheck2 phantom was calibrated with 6 MV beam where a 10 × 10 cm^2^ field was used to deliver 100 MU with the diode‐array at 7 cm depth. The machine was calibrated using the AAPM TG‐51 where 1 MU delivered by the machine represented 1 cGy at *d*_max_ = 1.5 cm using a 10 × 10 cm^2^ open field at SSD of 100 cm and reference temperature and pressure conditions. Then, the MapChecks2 phantom was cross‐calibrated with each electron energy using 10 × 10 cm^2^ cone and the diode‐array layer at 100 cm and top phantom cover removed. The electron dose measured by the MapCheck2 phantom is a relative dose and was used to compare with the dose calculated by the eMC in the same way it is usually used to measure photon doses to verify the calculated dose distributions from intensity‐modulation plans. The cross‐calibration between ion chamber to MapCheck2 and between the 6 MV to electron energies introduced dosimetric uncertainties within 2%. There are different factors that contribute to this uncertainty of the dose measurement with the MapCheck2: (a) the uncertainty in the output of the machine is within 1% which is verified with the monthly QA using the ADCL chamber (Exradin A12, Standard Imaging, Middleton, WI, USA) under reference conditions using the AAPM TG‐51,^38^ (b) cross‐calibration of the MapCheck2 with the Exradin ion chamber. The uncertainties in the electron doses distributions were quantified by comparison between the calculated and measured doses. The percentage differences between measured doses with MapCheck2 and calculated doses with eMC were given by the following formula: (*D*_measured_ − *D*_eMC_) ×100/*D*_measured_.

Heterogeneity correction was investigated both with measurement and calculation. The MapCheck2 phantom is made from different materials that include the solid water phantom encapsulating the diode‐array layer and the air cavities separating them. The MapCheck2 phantom was irradiated with open electron fields with different size 6 × 6 to 25 × 25 cm^2^ using energies ranging from 6 to 20 MeV. The corresponding dose distributions were calculated with eMC algorithm: (a) on the CT images of the MapCheck2 phantom using the uncorrected CT number values and (b) on the CT images of a solid water phantom that is water equivalent to the MapCheck2 phantom. Then, the measured dose distributions with the MapCheck2 phantom were compared with the calculated dose distributions with eMC. The measured dose distributions with the MapCheck2 phantom included directly the effects of heterogeneity of the different materials. The difference of the measured and calculated dose distributions provided direct test of the accuracy of the heterogeneity correction by the eMC algorithm. The difference between the calculated dose distributions on CT images of the MapCheck2 and solid water phantoms represented the effects of heterogeneity on dose calculation with eMC.

## 3 RESULTS

**FIGURE 2:**
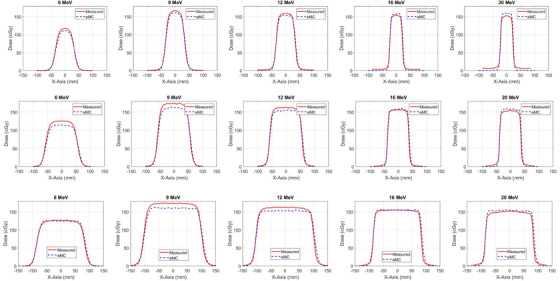
Dose profiles measured with the MapCheck2 phantom and the corresponding dose profiles calculated with electron Monte Carlo (eMC) algorithm for the electron energies 6–20 MeV and source‐to‐surface distance (SSD) = 110 cm in the different columns using different cones 6 × 6, 10 × 10, and 15 × 15 cm^2^ in the first, second, and third rows, respectively

**FIGURE 3:**
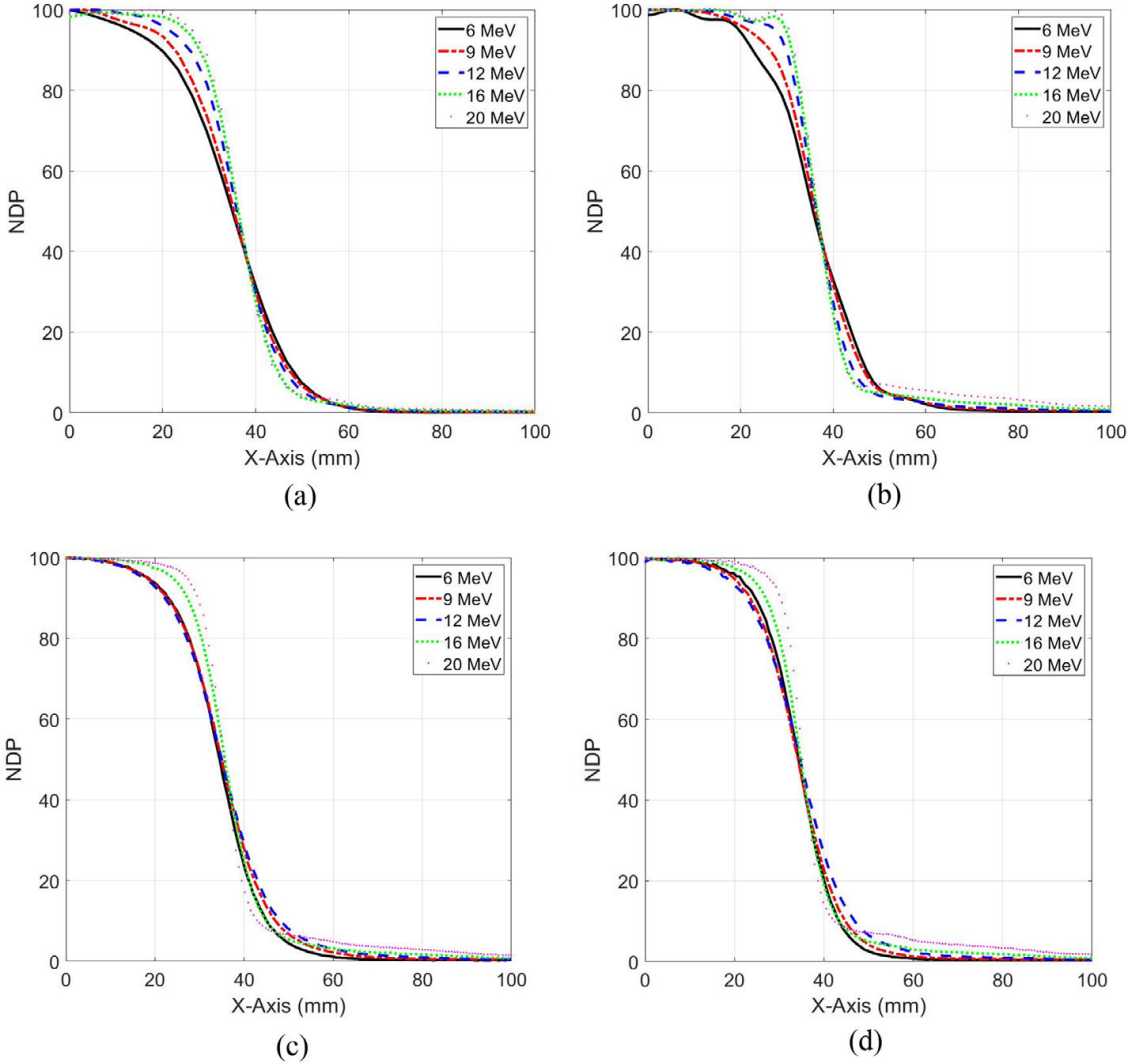
Comparison of the normalized dose profiles (NDP) (a) calculated with electron Monte Carlo (eMC) algorithm in solid water phantom, (b) measured with MapCheck2, (c) measured with the CC13 ion chamber in water, and (d) measured with the edge diode in water for electron energies 6, 9, 12, 16, and 20 MeV and source‐to‐surface distance (SSD) = 110 cm using the 6 × 6 cm^2^ cone

Figure 2 shows large dose discrepancies between the dose profiles calculated with eMC and the corresponding profiles measured with MapCheck2 particularly in the flat dose region around the central axis, in the penumbra (20%–80% isodose line), and in the umbra outside the field in the low dose level region (<20% isodose line). First, in the flat dose region, the calculated dose profiles had decreased dose deposition with local peaks and valleys in air cavities up to 8% in the encapsulation surrounding the diodes in the MapCheck2, while the measured dose distributions were mostly flat across the diodes and air cavities. The eMC calculation predicted lower dose level up to 3% in the flat dose region surrounding the central axis compared to dose level measured by MapCheck2 for lower electron energies 6, 12, and 9 MeV and higher dose levels up to 3% for higher electron energies 16 and 20 MeV. Second, the deviation in the dose deposition in the penumbra region resulted due to the uncertainty in the commissioning data measured with large detectors such as ion chambers (active volume = 0.13 cm^3^) that have limited resolution and caused volume averaging in the data input to the dose calculation algorithm. The MapCheck2 dose profiles were on the other hand measured with high resolution small diodes (0.8 mm) which showed sharper penumbra compared to the dose profiles measured with the ion chamber. Third, the increased dose deposition measured in the umbra region and in region outside the electron field resulted mostly from bremsstrahlung photons produced from energetic electrons in particular with energies equal and greater than 12 MeV as shown in Figure 3. The increased dose deposition at nearly 30 mm outside the field was not predicted by the eMC. In addition, the energy response of the diodes for low energy electrons contributed to the increased dose in the umbra mainly for low electron energies less than 12 MeV. This artifact resulted from the diodes enhanced response for low energy scattered electrons and photons in the dose profiles measured with MapCheck2 compared to the dose profiles measured with the ion chamber used in commissioning for treatment planning system. Figure 3 shows that the measured dose profiles using the CC13 ion chamber, edge diode (Sun Nuclear), and MapCheck2 agreed with each other and deviated from the corresponding dose profiled calculated with eMC. This indicated that enhanced dose deposition in the umbra region for high energy electron beams resulted mainly from laterally emitted bremsstrahlung and not due to the high response by the diodes.

**FIGURE 4:**
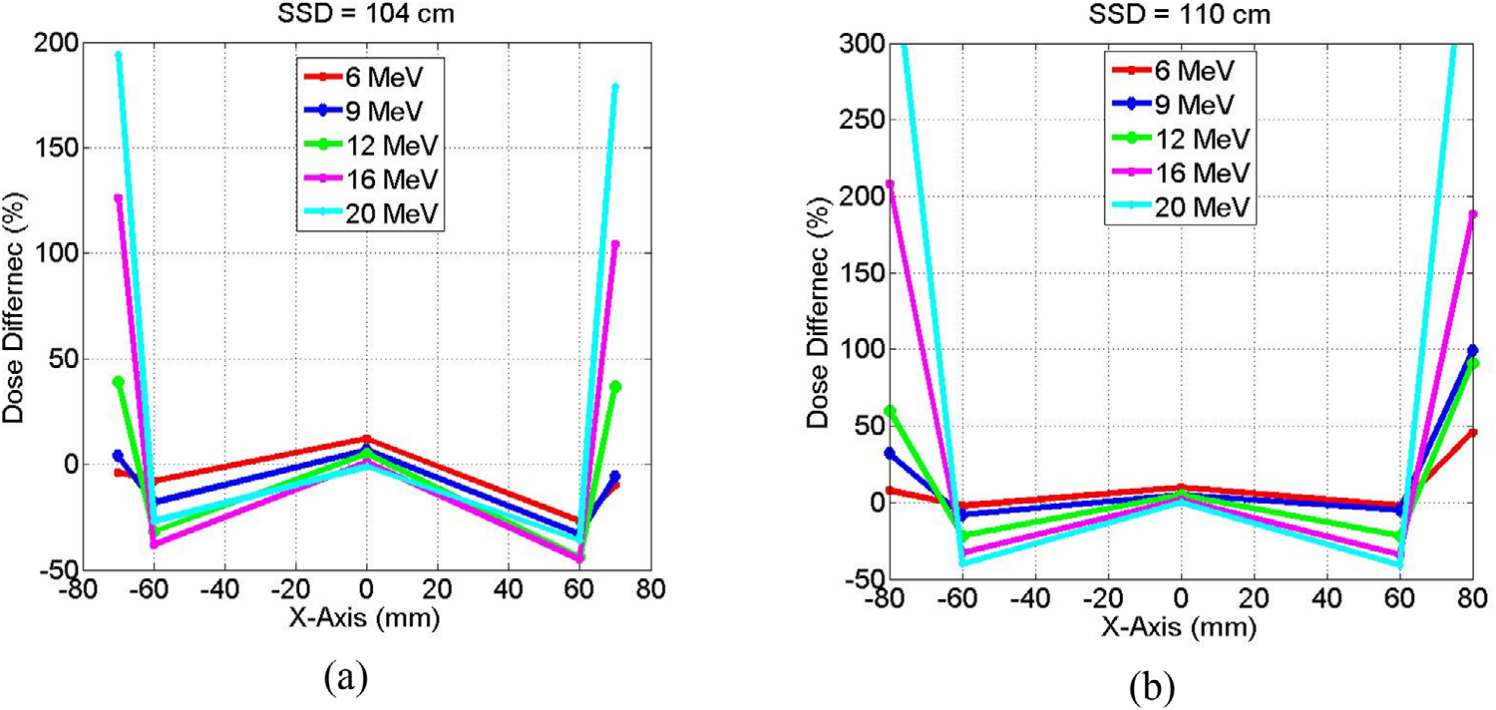
Comparison the percentage differences between measured doses with MapCheck2 and calculated doses with electron Monte Carlo (eMC) algorithm, (*D*_measured_ ‐ *D*_eMC_) × 100/*D*_measured_, at dose central axis (*x* = 0 mm), penumbra (*x* = +/‐60 mm), and umbra (*x* = +/‐80 mm) at (a) source‐to‐surface distance (SSD) = 104 cm and (b) SSD = 110 cm for a 10 × 10 cm^2^ field

Figure 4 shows that the eMC overestimated dose up to 40% for 20 MeV electrons compared to the measured dose with MapCheck2 in the penumbra region (*x* = +/‐60 mm). This was mostly due to lack of consideration of volume averaging in high dose gradient region of the ion chamber used in beam commissioning for eMC dose calculation algorithm. In the umbra region and outside the treatment field (*x* = +/‐80 mm), the eMC underestimated dose up to 300% compared to measured doses for 20 MeV electron beams mainly due to lack of consideration of bremsstrahlung emitted laterally and not accounted by eMC. These dose differences represented that local low dose deposition outside the treatment field in the surrounding normal tissue or nearby critical structures which were usually a small percentage of the high dose level at the central axis of the treatment field. The dose deposition in the umbra region had contributions from other sources that included the energetic electrons scattered laterally and deposited dose outside the electron field. In addition, high diode response for low energy electrons scattered laterally contributed to the dose in the umbra region (3%–5%). These contributions were nearly (0%–40%) as seen from low dose profiles of low energy electrons (6 and 9 MeV) compared to the substantial dose deposition from bremsstrahlung for high energy electrons (12–20 MeV) up to 300%. The eMC algorithms in the Eclipse treatment planning system did not account accurately for the lateral dose deposition outside the electron field.

**FIGURE 5:**
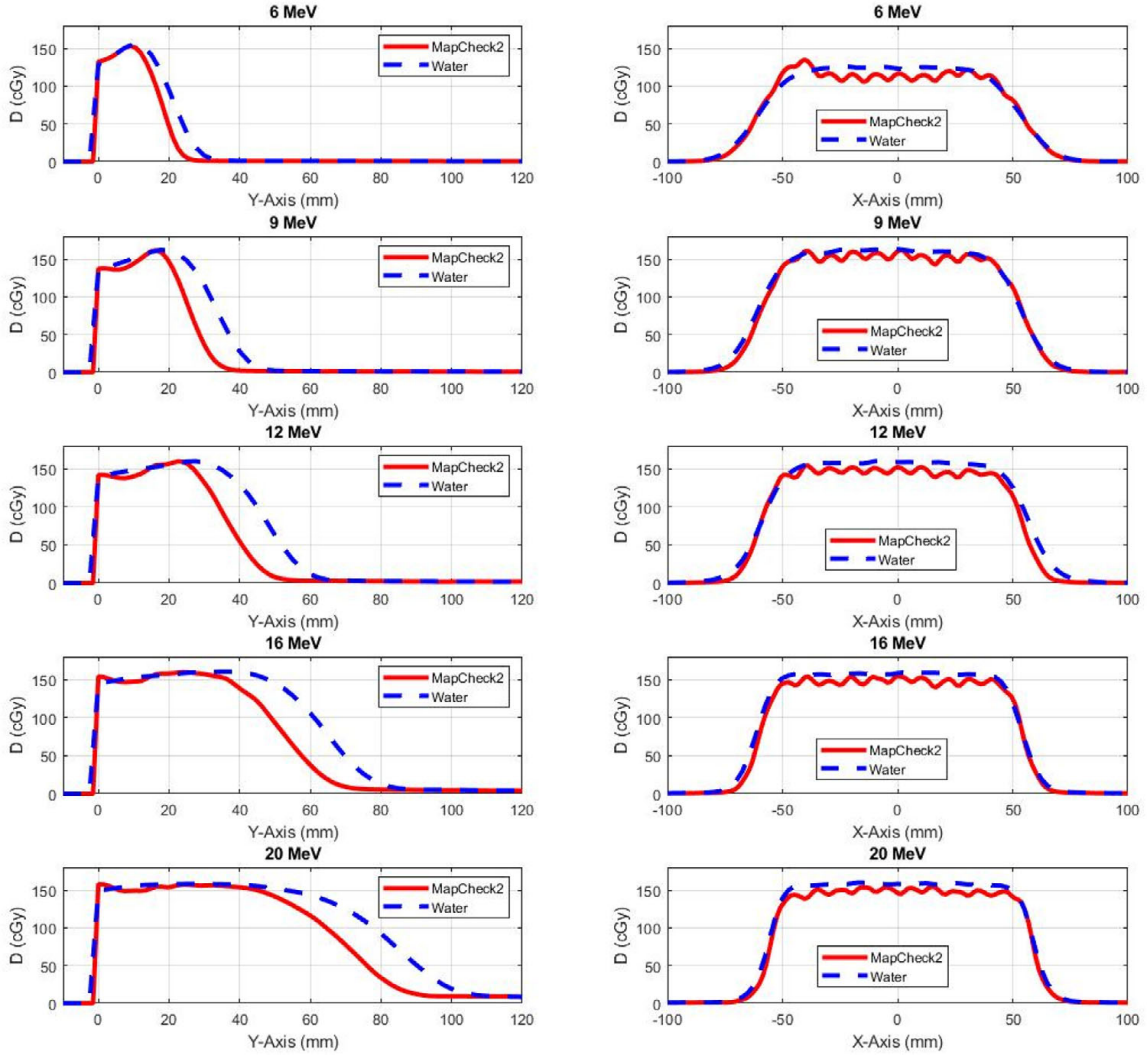
(a) Depth dose curves calculated by electron Monte Carlo (eMC) algorithm for electron energies (6–20 MeV) using the MapCheck2 phantom and a solid water phantom in the left column. (b) Dose profiles calculated by eMC for the different electron energies (6–20 MeV) in the MapCheck2 and solid water phantoms in the right column for 10 × 10 cm^2^ cone and source‐to‐surface distance (SSD) = 110 cm. The active layer including the diodes is located at 1 cm depth in the MapCheck2 where the diode and encapsulation extends about 5 mm

A large dose discrepancy in the calculated depth dose curves and dose profiles were observed in the MapCheck2 phantom with air and high‐Z material heterogeneities against water‐equivalent phantom as shown in Figure 5. The depth dose curves show that the entrance doses were higher in the MapCheck2 phantom than that in the water‐equivalent phantom (2.8%–5.7% for 6–20 MeV) due to metal artifacts in CT images from high‐Z composition of the MapCheck2. The electron ranges were larger in the solid water phantom than in the MapCheck2 due to its high‐Z composition. Depending on energy of the electron, the differences in range varied by 6–15 mm for 6–20 MeV, respectively. The calculated dose profiles show considerable discrepancies ranging 8%–10% between MapCheck2 and water‐equivalent phantoms due to MapCheck2 inhomogeneity around air cavities surrounding the diodes.

**FIGURE 6:**
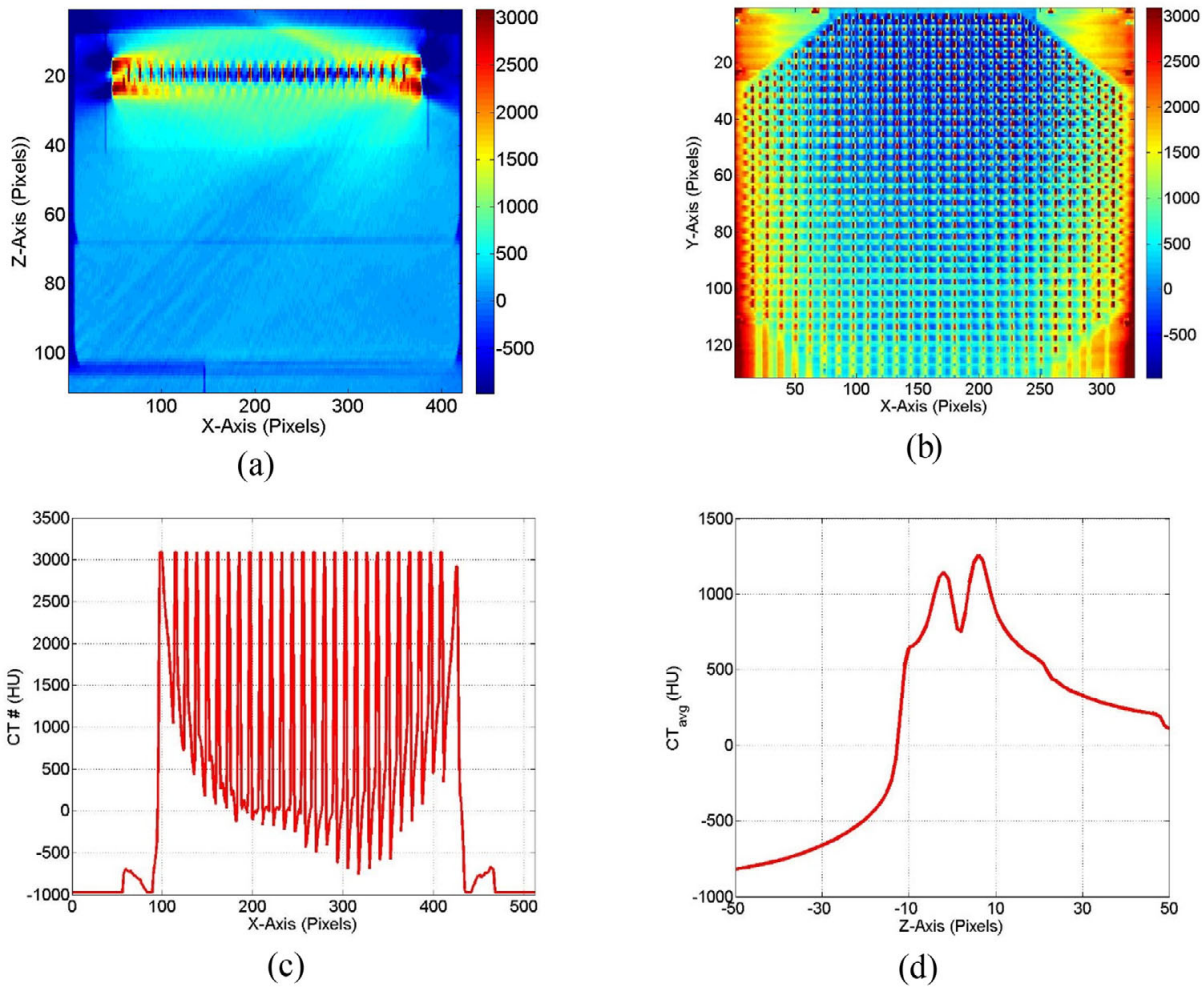
(a) Axial view of the CT number distributions of MapCheck2 multiple‐diode‐array detector from simulator CT imager where the CT values ranges nearly from ‐500 to 3096 HU due to image artifacts. (b) Coronal view of the CT number values of the MapCheck2 phantom. (c) The CT number profile along *x*‐axis across the layer that includes the diodes shown in (b) passing through the central diode. (d) Variation of the average CT numbers with distance perpendicular to the layer that includes the diode‐array (located at 0 pixel) in the MapCheck2 phantom. The average CT number values were calculated over a region of interest (100 × 100 pixels) in the middle of each layer

Figure 6a,b shows that the CT numbers of MapCheck2 phantom varied widely from ‐500 HU in air cavities between the diodes to nearly 3000 HU in the diodes and in the surrounding metal encapsulating the diodes. Figure 6c shows the profile CT numbers along *x*‐axis with large local variations due to composition of the diodes which correlated with the dose variation particularly in the flat region of profiles shown in Figure 5. The average CT number decreased with increasing distance from the effective detection layer that included the diode‐array (1200 HU) to nearly water equivalent (100 HU) at the surface of the phantom as shown in Figure 6d. There was a large decrease in the CT number values in the layer which included the air cavities between the diodes and then increase in the layers that included the diodes which are made from solid‐state material. The metal artifacts caused large deviations in CT numbers that led to large uncertainties in the extracted mass stopping power ratios used for dose calculation with eMC algorithm. These variations in the dose distributions resulted the local dose variations around the diodes in the dose distributions calculated with eMC considering heterogeneities of the MapCheck2 phantom as shown in the dose profiles in Figure 5b. However, these dose effects did not show up in the dose distributions measured with the MapCheck2 phantom as shown in Figure 2. Furthermore, these dose effects did not show up in the dose distributions calculated on the CT images of the solid water phantom that mimic the MapCheck2 phantom without heterogeneities as shown in Figure 5.

## 4 DISCUSSION

The eMC algorithm did not account well for the dose deposition laterally in the umbra region and outside the field. There were large dosimetric deviations between the calculated lateral dose deposition outside the field in the umbra region for high energy electrons and the measured dose profiles with MapCheck2. The major contribution to dose deposition in the umbra region resulted from the bremsstrahlung photons that were emitted laterally due to the interaction of the high energy electrons with phantom. The profiles acquired for the commissioning of the eMC dose calculation algorithm in the Eclipse treatment planning system were measured in air for a large field (40 × 40 cm^2^) where the bremsstrahlung production was very small even for high energy electron beams and thus it was not modeled appropriately with eMC. One approach to make the eMC model account for dose deposition emitted laterally is the inclusion of measured profiles for the different cones and energies in water that can be used to extract a radial correction factor. The eMC uses profiles that are measured in air for a large field of 40 × 40 cm^2^. These profiles were used to define the fluence profile before transport in the patient or medium. It seems that eMC was not able to model accurately the lateral dose deposition emitted by the bremsstrahlung that resulted from the interaction of high energy electrons with the medium using the air profile from the commissioning data.

The eMC overestimated the dose in the penumbra region. The use of an ion chamber in commissioning for measurements of electron beam profiles in air which caused volume averaging in high dose gradient region in the penumbra. Smaller detectors particularly diodes have high sensitivity and high spatial resolution which are recommended for the measurement of the electron dose profiles. In addition, appropriate dose measurement tools should be used for dose verification. In this study, the MapCheck2 was used to measure the 2D dose distributions from a wide range of fields with different cones and energies. The MapCheck2 provided a dosimetric tool with high sensitivity and high spatial resolution for dose verification from electron plans.

The uncertainties associated with heterogeneity corrections were investigated by dose calculated on the CT images of the MapCheck2 phantom relative to the dose calculated on the images of solid water phantom. The eMC algorithm was not able to handle the degradation of the CT number values in the CT images which were used in the Eclipse treatment planning system. The degradation in the CT number values resulted from the streaking artifacts induced by high‐Z materials in the MapCheck2 phantom. The uncertainties led to nearly 5% dose variations in the local dose regions surrounding the diodes where density of the medium changed from solid‐state materials to air cavities in the layer including the diodes which contradicted with the smooth dose profiles which were measured with the MapCheck2. The metal streaking artifacts affected the accuracy of the CT numbers in the phantom layers surrounding the diodes which caused uncertainties in the ranges of electron doses calculated for the MapCheck2 phantom compared to that in solid water phantom. The eMC algorithm used the CT number values to extract the density of the different materials for dose calculation. The CT numbers represented mainly the electron density that were used in the dose calculation algorithms for photon therapy where photon interactions with the medium resulted mainly from Compton scattering. However, the interaction of the electrons with the medium is mainly through Coulomb interactions with the electron shell and nucleus depending on the energy of the electron beam. Thus, the mass stopping power ratio has to be considered which represent actual tissue density and Z‐composition instead of electron density for correction of tissue heterogeneities in the eMC electron dose calculation algorithms.

In this study, the dose distributions were measured with MapCheck2 to verify the commissioning process and the dose distributions calculated with eMC. The MapCheck2 phantom provided a powerful dosimetric tool to measure 2D dose distributions and compare them directly with the calculated dose distributions by eMC. Response linearity, high sensitivity, high signal to noise ratio, and direct online readout of MapCheck2 were advantages to achieve the goals of this work. Furthermore, the MapCheck2 is made from different components that include solid water phantom, air cavities, and high‐Z materials used in this study to test heterogeneity correction by the eMC algorithm. However, the MapCheck2 phantom has limitations that include limited spatial resolution determined by interpolation process between the small diodes, and high‐Z materials which caused strong image artifacts in its CT images. The use of other detectors such as plane parallel ion chamber to measure the depth dose curves and small electron diode detectors to measure the profiles that can be scanned in a water phantom with heterogeneous objects might overcome some of the limitations of the MapCheck2 in a future study.

## 5 CONCLUSIONS

In this study, different dosimetric deviations between measured dose distributions and the corresponding dose distributions calculated with the eMC dose calculation algorithm in the Eclipse treatment planning system were investigated quantitatively. First, the lack of consideration of lateral dose deposition in the umbra region outside the field by bremsstrahlung that were produced by energetic electron beams resulted substantial dose uncertainties in the dose calculated by eMC. A second uncertainty was associated with the measurement of commissioning data needed for the eMC dose calculation algorithm that were acquired with large detectors such as ion chambers which caused volume averaging particularly in the penumbra regions with high dose gradients. Third, the use of inaccurate mass stopping ratios extracted from the CT number values for dose calculation resulted large dosimetric deviations between the dose distributions calculated using solid water phantoms and the MapCheck2 phantom with air and metal heterogeneities.

## CONFLICT OF INTEREST

None.
